# Mutations in the *NOT* Genes or in the Translation Machinery Similarly Display Increased Resistance to Histidine Starvation

**DOI:** 10.3389/fgene.2017.00061

**Published:** 2017-05-22

**Authors:** Martine A. Collart, Sari Kassem, Zoltan Villanyi

**Affiliations:** Department of Microbiology and Molecular Medicine, Centre Médical Universitaire (CMU), Faculty of Medicine, University of GenevaGeneva, Switzerland

**Keywords:** Ccr4-Not complex, histidine starvation, CNOT3, ribosome, translation, T-ALL leukemia

## Abstract

The *NOT* genes encode subunits of the conserved Ccr4-Not complex, a global regulator of gene expression, and in particular of mRNA metabolism. They were originally identified in a selection for increased resistance to histidine starvation in the yeast *S. cerevisiae*. Recent work indicated that the Not5 subunit, ortholog of mammalian CNOT3, determines global translation levels by defining binding of the Ccr4-Not scaffold protein Not1 to ribosomal mRNAs during transcription. This is needed for optimal translation of ribosomal proteins. In this work we searched for mutations in budding yeast that were resistant to histidine starvation using the same selection that originally led to the isolation of the *NOT* genes. We thereby isolated mutations in ribosome-related genes. This common phenotype of ribosome mutants and *not* mutants is in good agreement with the positive role of the Not proteins for translation. In this regard, it is interesting that frequent mutations in RPL5 and RPL10 or in CNOT3 have been observed to accumulate in adult T-cell acute lymphoblastic leukemia (T-ALL). This suggests that in metazoans a common function implicating ribosome subunits and CNOT3 plays a role in the development of cancer. In this perspective we suggest that the Ccr4-Not complex, according to translation levels and fidelity, could itself be involved in the regulation of amino acid biosynthesis levels. We discuss how this could explain why mutations have been identified in many cancers.

## Introduction

Cells use highly coordinated cascades of regulatory mechanisms to precisely define the production of specific gene products, which in turn determine development and differentiation, allow the cell to respond to the stressful environment or to adapt to new food sources. Sophisticated programs of gene expression integrate activities from multiple factors acting at different steps along the gene expression pathway. Each and every step of this pathway can be modulated, and many factors or protein complexes act at single steps, and in less frequent cases act at a couple of different steps. However, only one factor has been connected to most steps of the gene expression pathway, and this is the Ccr4-Not complex, which has recently been reviewed quite extensively (Collart and Timmers, 2004; Collart and Panasenko, 2012; Doidge et al., 2012; Miller and Reese, 2012; Collart, 2013; Collart et al., 2013; Panepinto et al., 2013; Reese, 2013; Wahle and Winkler, 2013; Winkler and Balacco, 2013; Chapat and Corbo, 2014; Inada and Makino, 2014; Panasenko, 2014; Shirai et al., 2014; Temme et al., 2014; Villanyi and Collart, 2015; Collart, 2016).

Ccr4-Not was first discovered in budding yeast (Denis, 1984; Collart and Struhl, 1993, 1994; Bai et al., 1999; Chen et al., 2001) where it is composed of 9 subunits, the Not1-Not5 proteins, Ccr4, and 3 Ccr4-associated factors, Caf1, Caf40, and Caf130. All of these subunits have orthologs in metazoans with the exception of Caf130. Metazoans have 2 orthologs for Caf1 called CNOT7 and CNOT8, 2 orthologs for Ccr4 called CNOT6 and CNOT6L and carry CNOT3 as a functional homolog of both Not5 and Not3, the products of a gene duplication event in budding yeast. Subunits with no yeast ortholog such as CNOT10 and CNOT11 are present in the metazoan Ccr4-Not complex (Albert et al., 2000). The metazoan ortholog of yeast Not4 is not a stable subunit of the metazoan Ccr4-Not complex but it does functionally complement the deletion of yeast Not4 (Albert et al., 2000; Bhaskar et al., 2015).

## Multiple activities of the Ccr4-Not complex

Two different enzymatic activities are associated with the Ccr4-Not complex: deadenylation and ubiquitination. Ccr4 and Caf1 mediate deadenylation (Tucker et al., 2001, 2002), the first and rate-limiting step for mRNA degradation in eukaryotes, while Not4, a RING E3 ligase, mediates ubiquitination (Albert et al., 2002). It poly-ubiquitinates and destabilizes some substrates (Cooper et al., 2012; Gronholm et al., 2012; Gulshan et al., 2012) and mono-ubiquitinates others without any consequence for their stability (Panasenko et al., 2006; Panasenko and Collart, 2012). Other non-enzymatic activities of the Ccr4-Not complex have been reported. For instance Not2, Not3 and Not5 are thought to promote decapping by interaction with the Pat1 protein (Maillet and Collart, 2002; Chen et al., 2014; Mathys et al., 2014; Rouya et al., 2014; Nishimura et al., 2015; Ozgur et al., 2015; Alhusaini and Coller, 2016). In contrast to these negative functions in expression of the genome, the Ccr4-Not complex also plays positive roles. For instance yeast Not5 promotes co-translational assembly of RNA Polymerase II (RNAPII) and SAGA, a function that correlates with the association of Not1 to relevant mRNAs (Villanyi et al., 2014; Kassem et al., 2017). Moreover, the Ccr4-Not complex can bind transcription elongation complexes and promote elongation of a backtracked RNAPII (Kruk et al., 2011). Recently an essential positive role of Not5 for production of the translation machine was uncovered. It is mediated by binding of Not1 to ribosomal mRNAs during their production in the nucleus (imprinting) (Gupta et al., 2016). Indeed, ribosomal protein mRNAs are enriched within the pool of mRNAs that can be immunopecipitated with Not1. The binding of Not1 to this category of mRNAs requires Not5 and negatively correlates with the level of these mRNAs in total extracts, but positively with their level in polysome fractions, with production of new ribosomal proteins and with global cellular translation levels. The fact that Not5 is needed in the nucleus to promote this Not1 binding to ribosomal mRNAs, and that Not1 binds to intronic sequences, indicated that Not1 was binding to newly produced mRNAs, hence the term “imprinting” (Gupta et al., 2016).

*In vivo* the deadenylase is functional when tethered to target mRNAs with the Not1 scaffold (reviewed in Collart, 2016). In contrast ubiquitination by Not4 does not always require its association with Not1 (reviewed in Collart, 2013). Tethering of the Not1 scaffold to mRNAs can also repress translation in a manner that is independent of any enzymatic activity of the complex. This is thought to occur via the interaction of the complex with proteins such as the eIF4E-binding proteins or the DDX6 RNA helicase (called Dhh1 in budding yeast).

It is intriguing that tethering of the Ccr4-Not machinery to mRNAs can promote translation and co-translational events (Villanyi et al., 2014; Gupta et al., 2016; Kassem et al., 2017) or promote mRNA silencing and degradation (Finoux and Seraphin, 2006). These opposite outcomes might be determined by the cellular compartment, in which Not1 initially binds mRNAs. Indeed it could be that the global architecture of the Ccr4-Not complex in the nucleus and the cytoplasm is different. Tethering of Not5 out of the yeast nucleus does not lead to co-depletion of nuclear Not1, supporting the idea that different Not1 complexes exist (Gupta et al., 2016). However, this issue still needs to be clarified and the role of the Not subunits in particular, associated with both repression and activation of gene expression, needs to be better defined.

## Ribosome mutants and *NOT* mutants are similarly resistant to histidine starvation

To consolidate our understanding of the functions mediated by the Not proteins we repeated the genetic selection in budding yeast that led to their isolation (Collart and Struhl, 1993). The idea was that we should isolate new mutations in the *NOT* genes, and potentially also additional mutations defining the cellular function affected by the Not proteins. We screened for new mutants that could grow on medium lacking histidine and containing 5 mM 3-aminotriazole (AT), a competitive inhibitor of the His3 enzyme (Collart and Struhl, 1993). His3 is necessary for yeast cells to produce histidine, and transcription of the *HIS3* gene, like other amino acid biosynthetic genes, is under the control of the Gcn4 transcriptional activator. In wild type cells the translation of Gcn4 is regulated by the presence of upstream open reading frames (ORFs) and its production increases in response to starvation. For the selection, we used a starting strain (*MAT*a *ura3-52 trp1-*Δ*1 leu2-PET56 gal2 gcn4-*Δ*1*; Hope and Struhl, 1986) with a deletion of the endogenous *GCN4*, carrying a plasmid expressing a mutant Gcn4 (YCp88-Sc4363) with a truncated activation domain (Hope and Struhl, 1986) and expressed from the constitutive *DED1* promoter lacking uORFs (Collart and Struhl, 1993).

From the new recessive mutants isolated that were resistant to 5 mM AT in the growth medium, 9 strains carried mutations in *NOT1*, 2 in *NOT2*, 14 in *NOT3*, and 2 in *NOT4*. The mutations isolated in *NOT5* have been described (Oberholzer and Collart, 1998). Fifteen other recessive mutants had slow growth or temperature sensitive phenotypes that co-segregated with AT-resistance. Surprisingly they defined 15 different complementation groups. We isolated clones complementing the mutations for 6 of these and sequenced the clone ends to identify the genomic fragments. Each clone carried either a ribosomal protein gene or a gene important for ribosome biogenesis. To determine whether these mutants were defective in ribosome biogenesis, we analyzed the polysome profiles of the 6 mutants by fractionation of total cellular extracts on a sucrose gradient. All 6 had defective polysome profiles, even at the permissive temperature (data not shown). In particular shoulders indicative of the presence of half-mers were visible in all mutant profiles (see below). This indicated that ribosomes were altered, and consistently, mutations were identified for each mutant in the ribosome-related gene. Sub-clones carrying these genes, but not sub-clones lacking these genes, complemented the mutant phenotypes (Table 1).

**Table 1:** **List of strains and mutations isolated in the selection for AT-resistance**.

**Strain**	**Gene**	**Protein function**	**Mutation**	**Codon**	**mutation**
You95	*RPL33A*	Ribosomal	A553T	10	Nonsense
You69	*FHL1*	Transcription factor for ribosomal protein genes	G1993Δ	665	Frameshift Stop at 674
You90	*RIX1*	35S processing	C295T	99	Nonsense
You101	*TSR4*	20S processing	C684A	228	Nonsense
You61	*RPL28*	Ribosomal	G681T	57	G to V
You114	*RPL10*	Ribosomal	G481T	161	G to V

To confirm that the identified mutations were responsible for AT-resistance we focused on *RPL10*, integrated a *URA3* marker gene at the *RPL10* locus and confirmed by crosses and tetrad dissection that it co-segregated with the mutant phenotype. We also recovered the mutant gene on a plasmid and confirmed that it could not complement the temperature sensitive growth phenotype. Hence, mutations in genes that impair ribosome biogenesis and decrease global translation, like mutations in the *NOT* genes, lead to resistance to histidine starvation.

## Specific Not1 binding responds to decreased protein synthesis

*HIS3* mRNA is increased in *not* mutants (Collart and Struhl, 1993, 1994; Oberholzer and Collart, 1998) and we observed a similar increase in the *rpl10* mutant (data not shown). This most likely contributes to AT-resistance, possibly together with an increase in free amino acids due to reduced translation. These findings indicate that defective or reduced protein synthesis, as observed in *not* mutants or in ribosome-related mutants, is connected, possibly by the means of a cellular signal, to a relative increase in the transcript levels of an amino acid biosynthesis gene, namely *HIS3*. This raises the question of what the nature of the signal is. This signal cannot be the well-established eIF2α phosphorylation and translational up-regulation of Gcn4, known to respond to amino acid starvation, since the strain used in our selection expresses a Gcn4 derivative without uORFs.

**Figure 1:**
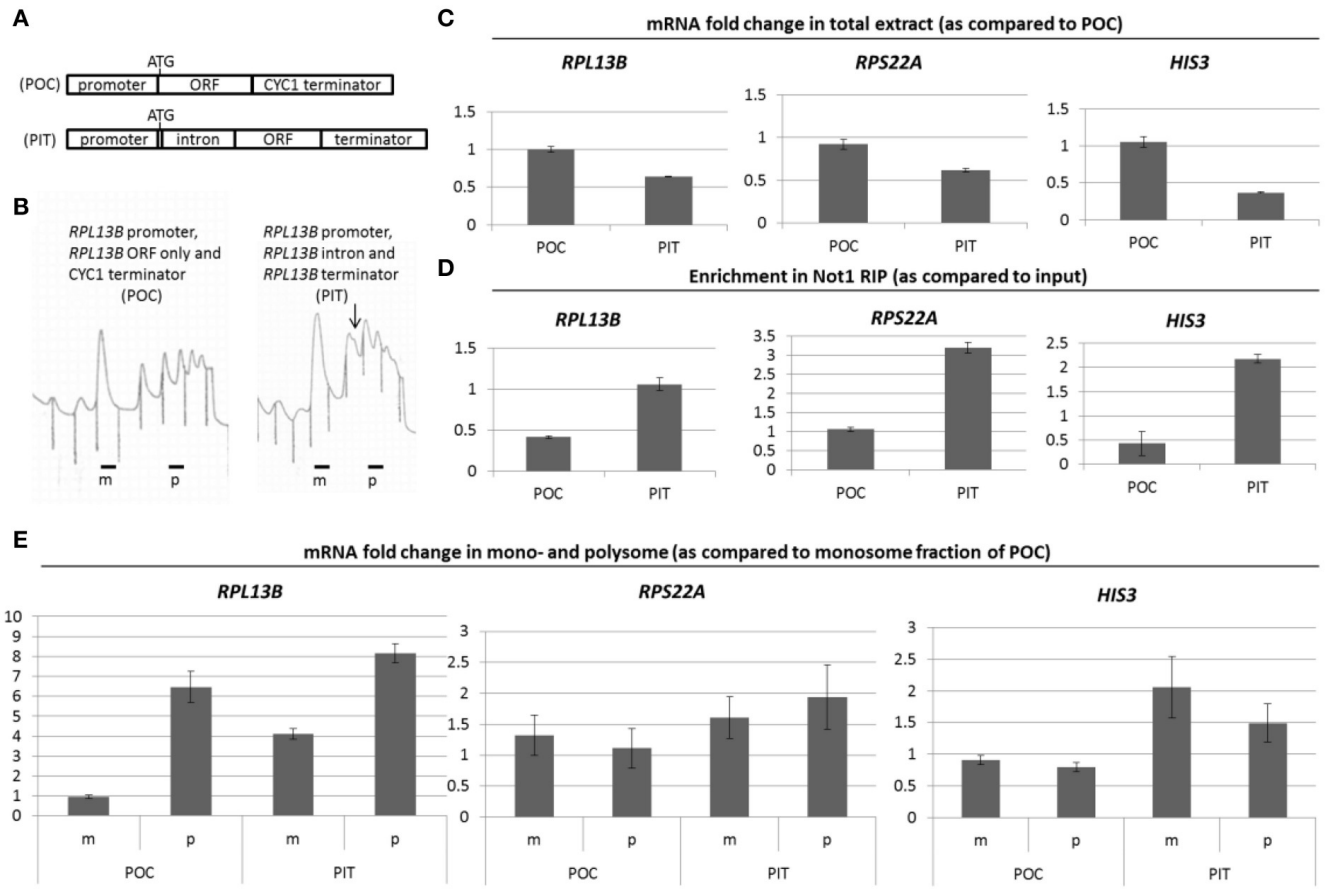
**(A)** *RPL13B* constructs. Similarly to the majority of yeast RP genes with introns, in the case of *RPL13B* the entire ORF is encoded in the 2nd exon except for a methionine which is encoded in the 1st exon. Construct “PIT” contains all the endogenous elements of the *RPL13B* gene. The intron is removed and the terminator is changed to a *CYC1* terminator in “POC.” **(B)** Extracts from cells expressing PIT or POC were separated on a sucrose gradient to follow the polysome profiles. The arrow points to a shoulder on the disome peak. **(C,D)** Levels of the indicated mRNAs were measured in total extracts **(C)** or in Not1 immunoprecipitates **(D)**. **(E)** Levels of the indicated mRNAs were measured in the monosome (m) and polysome (p) fractions as previously described (Gupta et al., 2016). The specific mRNAs measured are *RPL13B, RPS22A*, and *HIS3* as indicated, and normalization was to the *NIP1* mRNA levels.

We considered the possibility that the Not proteins themselves were part of the signaling pathway, since amino acid biosynthesis gene products are amongst the most up-regulated newly produced proteins in *not5*Δ (Gupta et al., 2016). Moreover, Not1 is significantly less associated with *HIS3* mRNA in *not5*Δ, and *HIS3* mRNA levels increase in total extracts and in polysomes (Gupta et al., 2016). To test this idea, we chose to compare 2 isogenic strains that had as only difference that they expressed a randomly chosen ribosomal protein gene at different levels. We prepared 2 *rpl13b*Δ stains containing plasmids expressing Rpl13b at different levels. One plasmid carried the endogenous *RPL13B* gene (promoter, intron, and terminator: PIT), and one plasmid contained the *RPL13B* promoter, open reading frame (ORF), and the heterologous *CYC1* terminator (POC). It also lacked an intron (constructs depicted on Figure 1A). Cells expressing PIT had higher levels of polysomes compared to POC, but the polysomes had shoulders indicative of the presence of ribosome half-mers and ribosome biogenesis defects (Figure 1B). Indeed, in a normal polysome profile the identified peaks indicate one extra ribosome per mRNA (dimer, trimer, tetramer…). Half-mers instead indicate an unequal number of 40S and 60S ribosomal subunits on the mRNA. Expression of *RPL13B* from PIT was lower than from POC (Figure 1C, left panel). This different expression of Rpl13b induced differences in relative expression of other cellular mRNAs such as other ribosomal mRNAs (e.g., *RPS22A)* or *HIS3* (Figure 1C, middle and right panels). This correlated with changes in relative binding of Not1 to those mRNAs in the 2 strains (Figure 1D) and in relative presence of those mRNAs in monosomes and polysomes (Figure 1E). *NIP1* mRNA, whose association with Not1 does not change with different expression of ribosomal mRNAs (Gupta et al., 2016), was used for normalization.

Hence changes in ribosome production lead to modification of Not1 binding to *HIS3* and ribosomal mRNAs, and to changes in relative translation of those mRNAs.

## Resistance to starvation resulting from ribosome or Not protein mutations could explain their frequent occurrence in cancer

This study reveals that mutations in yeast ribosomal genes or ribosome biogenesis genes, like mutations in the *NOT* genes that affect global translation (Gupta et al., 2016), lead to increased resistance to histidine starvation. In metazoan cells, altering Not function or disrupting the translation machine, can also have similar phenotypes. Indeed, mutations in CNOT3, the ortholog of Not5, or in the RPL5 and RPL10 ribosomal proteins, are associated with adult T-cell acute lymphoblastic leukemia (T-ALL) (De Keersmaecker et al., 2013). T-ALL is an aggressive malignancy caused by the accumulation of genomic lesions leading to altered gene dosage. RPL5 and RPL10 occupy neighboring positions in the 60S subunit of the ribosomes, next to the central protuberance, which is in close vicinity to both the P, A, and E sites of the ribosomes. Around 8% of pediatric T-ALL patients harbor an Arg98Ser mutation in RPL10, which was shown to reduce translation fidelity (Sulima et al., 2014). RPL22 on the other hand forms the narrowest constriction of the ribosomal exit tunnel (Nakatogawa and Ito, 2002). Mutations in RPL22 are believed to alter protein synthesis efficacy. CNOT3 in T-ALL patients frequently carries missense mutations in Arg57 that affects splicing and reduces CNOT3 levels (De Keersmaecker et al., 2013). According to our work in yeast this should lead to reduced global translation and also to defects in co-translational complex assembly and consequently to elevated protein aggregation (Panasenko and Collart, 2012; Villanyi et al., 2014; Gupta et al., 2016; Kassem et al., 2017). Thus it appears that alteration of the process of translation in cancer cells can ensure their survival.

Numerous reports have described the presence of frequent mutations in ribosome subunits in tumor cells and different mechanisms have been considered to explain how RP mutations contribute to tumorigenesis or tumor maintenance (reviewed in Ruggero, 2013; Wang et al., 2015; Goudarzi and Lindstrom, 2016). Three main pathways have generally been considered: global suppression of protein synthesis, specific suppression of protein synthesis or finally extra-ribosomal functions. Our current observations suggest that relative up-regulation of amino acid biosynthesis genes resulting from global alteration of the translation process might contribute to sustain growth of the tumor cells. Since Not5 in particular appears to be involved in the regulatory loop linking translation to amino acid biosynthesis, the ortholog CNOT3 may have a tumor suppressor function. This is certainly compatible with the fact that mutations in CNOT3 have been identified in many tumors (Collart et al., 2013).

It is important to note that an impact of CNOT3 on amino acid biosynthesis is unlikely to be the only mechanism, by which a mutation of CNOT3 in tumor cells might contribute to tumor survival. This protein has very broad cellular functions and it has for instance been described as a modifier of gene expression that leads to incomplete penetrance of PRFP31 mutations in retinitis pigmentosa (Venturini et al., 2012). We have reported that in yeast Not1 and Not5 are important for gene expression homeostasis, buffering between transcription, translation and mRNA decay to maintain steady state protein levels (Villanyi and Collart, 2015). The deletion of Not5 leads to very slow growth of yeast cells, and the deletion of both alleles of CNOT3 is embryonic lethal in mice. Moreover, CNOT3^+/−^ mice have many disturbed physiological functions (Morita et al., 2011; Shirai et al., 2014) indicating a very essential function of this protein (reviewed in Collart et al., 2013). It could be that the lower dose of *CNOT3* in T-ALL cells does not disturb the major gene expression buffering function of CNOT3, but does have an impact on protein folding and complex assembly. The same could be true for the frequent *RPL5, RPL10*, and *RPL22* mutations: affecting translation fidelity or efficacy has obvious dosage compensatory effects, as improperly assembled or folded proteins are likely to be non-functional, and prone to degradation or aggregation. In this context it is interesting to note that it has been reported that null mutants of specific Ccr4-Not subunits reduce the viability of aneuploid yeast cells (Tange et al., 2012). There may be a very fine balance between the impairment of Ccr4-Not function that can ensure survival of tumor cells with genomic lesions, or instead more severe *ccr4-not* mutations that will be toxic.

## Conclusion

The Ccr4-Not complex acts at all stages of the gene expression pathway, and has both positive and negative effects on gene expression. Clarifying which contribution of Ccr4-Not regulation is important or lost in specific biological contexts is a real challenge. Work *in vivo* cannot easily distinguish direct from indirect effects, whereas studies *in vitro* define what the complex can do, but not how and when this is relevant *in vivo*. Here we point out that perturbation of translation is positively affecting amino acid biosynthesis that can facilitate cancer cell survival. We suggest that Not5 might not only regulate production of the translation machinery but also participate in the cross-talk between translation and amino acid production. Finally, we put forward the idea that impaired translation might contribute to dosage compensation via production of non-functional proteins. As the story of Ccr4-Not unfolds, it is clear that its understanding requires an open mind.

## Author contributions

MC contributed to experimental design, interpretation, and writing. SK contributed to interpretation and writing. ZV contributed to experimental work, interpretation, and writing.

## Funding

This work was supported by grant [31003a_135794] from the Swiss National Science Foundation awarded to MC.

### Conflict of interest statement

The authors declare that the research was conducted in the absence of any commercial or financial relationships that could be construed as a potential conflict of interest.

## References

[B1] Albert T. K. Hanzawa H. Legtenberg Y. I. de Ruwe M. J. van den Heuvel F. A. Collart M. A.. (2002). Identification of a ubiquitin-protein ligase subunit within the CCR4-NOT transcription repressor complex. EMBO J. 21, 355–364. 10.1093/emboj/21.3.355 11823428 PMC125831

[B2] Albert T. K. Lemaire M. van Berkum N. L. Gentz R. Collart M. A. Timmers H. T. (2000). Isolation and characterization of human orthologs of yeast CCR4-NOT complex subunits. Nucleic Acids Res. 28, 809–817. 10.1093/nar/28.3.809 10637334 PMC102560

[B3] Alhusaini N. Coller J. (2016). The deadenylase components Not2p, Not3p, and Not5p promote mRNA decapping. RNA 22, 709–721. 10.1261/rna.054742.115 26952104 PMC4836645

[B4] Bai Y. Salvadore C. Chiang Y. C. Collart M. A. Liu H. Y. Denis C. L. (1999). The CCR4 and CAF1 proteins of the CCR4-NOT complex are physically and functionally separated from NOT2, NOT4, and NOT5. Mol. Cell. Biol. 19, 6642–6651. 10.1128/MCB.19.10.6642 10490603 PMC84645

[B5] Bhaskar V. Basquin J. Conti E. (2015). Architecture of the ubiquitylation module of the yeast Ccr4-Not complex. Structure 23, 921–928. 10.1016/j.str.2015.03.011 25914052 PMC4431670

[B6] Chapat C. Corbo L. (2014). Novel roles of the CCR4-NOT complex. Wiley Interdiscip. Rev. RNA 5, 883–901. 10.1002/wrna.1254 25044499

[B7] Chen J. Rappsilber J. Chiang Y. C. Russell P. Mann M. Denis C. L. (2001). Purification and characterization of the 1.0 MDa CCR4-NOT complex identifies two novel components of the complex. J. Mol. Biol. 314, 683–694. 10.1006/jmbi.2001.5162 11733989

[B8] Chen Y. Boland A. Kuzuoğlu-Öztürk D. Bawankar P. Loh B. Chang C. T.. (2014). A DDX6-CNOT1 complex and W-binding pockets in CNOT9 reveal direct links between miRNA target recognition and silencing. Mol. Cell 54, 737–750. 10.1016/j.molcel.2014.03.034 24768540

[B9] Collart M. A. (2013). The NOT4 RING E3 ligase: a relevant player in co-translational quality control. ISRN Mol. Biol. 2013:548359. 10.1155/2013/548359 27335678 PMC4890865

[B10] Collart M. A. (2016). The Ccr4-Not complex is a key regulator of eukaryotic gene expression. Wiley Interdiscip. Rev. RNA, 7, 438–454. 10.1002/wrna.1332 26821858 PMC5066686

[B11] Collart M. A. Panasenko O. O. (2012). The Ccr4–not complex. Gene 492, 42–53. 10.1016/j.gene.2011.09.033 22027279

[B12] Collart M. A. Panasenko O. O. Nikolaev S. I. (2013). The Not3/5 subunit of the Ccr4-Not complex: a central regulator of gene expression that integrates signals between the cytoplasm and the nucleus in eukaryotic cells. Cell. Signal. 25, 743–751. 10.1016/j.cellsig.2012.12.018 23280189

[B13] Collart M. A. Struhl K. (1993). CDC39, an essential nuclear protein that negatively regulates transcription and differentially affects the constitutive and inducible HIS3 promoters. EMBO J. 12, 177–186. 8428577 10.1002/j.1460-2075.1993.tb05643.x PMC413189

[B14] Collart M. A. Struhl K. (1994). NOT1(CDC39), NOT2(CDC36), NOT3, and NOT4 encode a global-negative regulator of transcription that differentially affects TATA-element utilization. Genes Dev. 8, 525–537. 10.1101/gad.8.5.525 7926748

[B15] Collart M. A. Timmers H. T. (2004). The eukaryotic Ccr4-not complex: a regulatory platform integrating mRNA metabolism with cellular signaling pathways? Prog. Nucleic Acid Res. Mol. Biol. 77, 289–322. 10.1016/S0079-6603(04)77008-7 15196896

[B16] Cooper K. F. Scarnati M. S. Krasley E. Mallory M. J. Jin C. Law M. J.. (2012). Oxidative-stress-induced nuclear to cytoplasmic relocalization is required for Not4-dependent cyclin C destruction. J. Cell Sci. 125, 1015–1026. 10.1242/jcs.096479 22421358 PMC3311932

[B17] De Keersmaecker K. Atak Z. K. Li N. Vicente C. Patchett S. Girardi T.. (2013). Exome sequencing identifies mutation in CNOT3 and ribosomal genes RPL5 and RPL10 in T-cell acute lymphoblastic leukemia. Nat. Genet. 45, 186–190. 10.1038/ng.2508 23263491 PMC5547913

[B18] Denis C. L. (1984). Identification of new genes involved in the regulation of yeast alcohol dehydrogenase II. Genetics 108, 833–834. 6392016 10.1093/genetics/108.4.833 PMC1224268

[B19] Doidge R. Mittal S. Aslam A. Winkler G. S. (2012). Deadenylation of cytoplasmic mRNA by the mammalian Ccr4-Not complex. Biochem. Soc. Trans. 40, 896–901. 10.1042/BST20120074 22817755

[B20] Finoux A. L. Seraphin B. (2006). *In vivo* targeting of the yeast Pop2 deadenylase subunit to reporter transcripts induces their rapid degradation and generates new decay intermediates. J. Biol. Chem. 281, 25940–25947. 10.1074/jbc.M600132200 16793769

[B21] Goudarzi K. M. Lindstrom M. S. (2016). Role of ribosomal protein mutations in tumor development (Review). Int. J. Oncol. 48, 1313–1324. 10.3892/ijo.2016.3387 26892688 PMC4777597

[B22] Gronholm J. Kaustio M. Myllymäki H. Kallio J. Saarikettu J. Kronhamn J.. (2012). Not4 enhances JAK/STAT pathway-dependent gene expression in Drosophila and in human cells. FASEB J. 26, 1239–1250. 10.1096/fj.11-195875 22159038

[B23] Gulshan K. Thommandru B. Moye-Rowley W. S. (2012). Proteolytic degradation of the Yap1 transcription factor is regulated by subcellular localization and the E3 ubiquitin ligase Not4. J. Biol. Chem. 287, 26796–26805. 10.1074/jbc.M112.384719 22707721 PMC3411017

[B24] Gupta I. Villanyi Z. Kassem S. Hughes C. Panasenko O. O. Steinmetz L. M.. (2016). Translational capacity of a cell is determined during transcription elongation via the Ccr4-not complex. Cell Rep. 15, 1782–1794. 10.1016/j.celrep.2016.04.055 27184853 PMC4880543

[B25] Hope I. Struhl K. (1986). Functional dissection of a eukaryotic transcriptional activator protein. Cell 46, 885–894. 10.1016/0092-8674(86)90070-X 3530496

[B26] Inada T. Makino S. (2014). Novel roles of the multi-functional CCR4-NOT complex in post-transcriptional regulation. Front. Genet. 5:135. 10.3389/fgene.2014.00135 24904636 PMC4033010

[B27] Kassem S. Villanyi Z. Collart M. A. (2017). Not5-dependent co-translational assembly of Ada2 and Spt20 is essential for functional integrity of SAGA. Nucleic Acids Res. 45, 1186–1199. 10.1093/nar/gkw1059 28180299 PMC5388395

[B28] Kruk J. A. Dutta A. Fu J. Gilmour D. S. Reese J. C. (2011). The multifunctional Ccr4-Not complex directly promotes transcription elongation. Genes Dev. 25, 581–593. 10.1101/gad.2020911 21406554 PMC3059832

[B29] Maillet L. Collart M. A. (2002). Interaction between Not1p, a component of the Ccr4-not complex, a global regulator of transcription, and Dhh1p, a putative RNA helicase. J. Biol. Chem. 277, 2835–2842. 10.1074/jbc.M107979200 11696541

[B30] Mathys H. Basquin J. Ozgur S. Czarnocki-Cieciura M. Bonneau F. Aartse A.. (2014). Structural and biochemical insights to the role of the CCR4-NOT complex and DDX6 ATPase in microRNA repression. Mol. Cell 54, 751–765. 10.1016/j.molcel.2014.03.036 24768538

[B31] Miller J. E. Reese J. C. (2012). Ccr4-Not complex: the control freak of eukaryotic cells. Crit. Rev. Biochem. Mol. Biol. 47, 315–333. 10.3109/10409238.2012.667214 22416820 PMC3376659

[B32] Morita M. Oike Y. Nagashima T. Kadomatsu T. Tabata M. Suzuki T.. (2011). Obesity resistance and increased hepatic expression of catabolism-related mRNAs in *Cnot3*+*/*− mice. EMBO J. 30, 4678–4691. 10.1038/emboj.2011.320 21897366 PMC3243589

[B33] Nakatogawa H. Ito K. (2002). The ribosomal exit tunnel functions as a discriminating gate. Cell 108, 629–636. 10.1016/S0092-8674(02)00649-9 11893334

[B34] Nishimura T. Padamsi Z. Fakim H. Milette S. Dunham W. H. Gingras A. C.. (2015). The eIF4E-binding protein 4E-T Is a component of the mRNA decay machinery that bridges the 5' and 3' termini of target mRNAs. Cell Rep. 11, 1425–1436. 10.1016/j.celrep.2015.04.065 26027925

[B35] Oberholzer U. Collart M. A. (1998). Characterization of NOT5 that encodes a new component of the Not protein complex. Gene 207, 61–69. 10.1016/S0378-1119(97)00605-7 9511744

[B36] Ozgur S. Basquin J. Kamenska A. Filipowicz W. Standart N. Conti E. (2015). Structure of a human 4E-T/DDX6/CNOT1 complex reveals the different interplay of DDX6-binding proteins with the CCR4-NOT complex. Cell Rep. 13, 703–711. 10.1016/j.celrep.2015.09.033 26489469

[B37] Panasenko O. O. (2014). The role of the E3 ligase Not4 in cotranslational quality control. Front. Genet. 5:141. 10.3389/fgene.2014.00141 24904641 PMC4032911

[B38] Panasenko O. O. Collart M. A. (2012). Presence of Not5 and ubiquitinated Rps7A in polysome fractions depends upon the Not4 E3 ligase. Mol. Microbiol. 83, 640–653. 10.1111/j.1365-2958.2011.07957.x 22243599

[B39] Panasenko O. Landrieux E. Feuermann M. Finka A. Paquet N. Collart M. A. (2006). The yeast Ccr4-Not complex controls ubiquitination of the nascent-associated polypeptide (NAC-EGD) complex. J. Biol. Chem. 281, 31389–31398. 10.1074/jbc.M604986200 16926149

[B40] Panepinto J. C. Heinz E. Traven A. (2013). The cellular roles of Ccr4-NOT in model and pathogenic fungi-implications for fungal virulence. Front. Genet. 4:302. 10.3389/fgene.2013.00302 24391665 PMC3868889

[B41] Reese J. C. (2013). The control of elongation by the yeast Ccr4-not complex. Biochim. Biophys. Acta 1829, 127–133. 10.1016/j.bbagrm.2012.09.001 22975735 PMC3545033

[B42] Rouya C. Siddiqui N. Morita M. Duchaine T. F. Fabian M. R. Sonenberg N. (2014). Human DDX6 effects miRNA-mediated gene silencing via direct binding to CNOT1. RNA 20, 1398–1409. 10.1261/rna.045302.114 25035296 PMC4138323

[B43] Ruggero D. (2013). Translational control in cancer etiology. Cold Spring Harb. Perspect. Biol. 5:a012336. 10.1101/cshperspect.a012336 22767671 PMC3552512

[B44] Shirai Y. T. Suzuki T. Morita M. Takahashi A. Yamamoto T. (2014). Multifunctional roles of the mammalian CCR4-NOT complex in physiological phenomena. Front. Genet. 5:286. 10.3389/fgene.2014.00286 25191340 PMC4139912

[B45] Sulima S. O. Patchett S. Advani V. M. De Keersmaecker K. Johnson A. W. Dinman J. D. (2014). Bypass of the pre-60S ribosomal quality control as a pathway to oncogenesis. Proc. Natl. Acad. Sci. U.S.A. 111, 5640–5645. 10.1073/pnas.1400247111 24706786 PMC3992666

[B46] Tange Y. Kurabayashi A. Goto B. Hoe K. L. Kim D. U. Park H. O.. (2012). The CCR4-NOT complex is implicated in the viability of aneuploid yeasts. PLoS Genet. 8:e1002776. 10.1371/journal.pgen.1002776 22737087 PMC3380822

[B47] Temme C. Simonelig M. Wahle E. (2014). Deadenylation of mRNA by the CCR4-NOT complex in Drosophila: molecular and developmental aspects. Front. Genet. 5:143. 10.3389/fgene.2014.00143 24904643 PMC4033318

[B48] Tucker M. Staples R. R. Valencia-Sanchez M. A. Muhlrad D. Parker R. (2002). Ccr4p is the catalytic subunit of a Ccr4p/Pop2p/Notp mRNA deadenylase complex in *Saccharomyces cerevisiae*. EMBO J. 21, 1427–1436. 10.1093/emboj/21.6.1427 11889048 PMC125913

[B49] Tucker M. Valencia-Sanchez M. A. Staples R. R. Chen J. Denis C. L. Parker R.. (2001). The transcription factor associated Ccr4 and Caf1 proteins are components of the major cytoplasmic mRNA deadenylase in *Saccharomyces cerevisiae*. Cell 104, 377–386. 10.1016/S0092-8674(01)00225-2 11239395

[B50] Venturini G. Rose A. M. Shah A. Z. Bhattacharya S. S. Rivolta C. (2012). CNOT3 is a modifier of PRPF31 mutations in retinitis pigmentosa with incomplete penetrance. PLoS Genet. 8:e1003040. 10.1371/journal.pgen.1003040 23144630 PMC3493449

[B51] Villanyi Z. Collart M. A. (2015). Ccr4-Not is at the core of the eukaryotic gene expression circuitry. Biochem. Soc. Trans. 43, 1253–1258. 10.1042/BST20150167 26614669

[B52] Villanyi Z. Ribaud V. Kassem S. Panasenko O. O. Pahi Z. Gupta I.. (2014). The Not5 subunit of the ccr4-not complex connects transcription and translation. PLoS Genet. 10:e1004569. 10.1371/journal.pgen.1004569 25340856 PMC4207488

[B53] Wahle E. Winkler G. S. (2013). RNA decay machines: deadenylation by the Ccr4-Not and Pan2-Pan3 complexes. Biochim. Biophys. Acta 1829, 561–570. 10.1016/j.bbagrm.2013.01.003 23337855

[B54] Wang W. Nag S. Zhang X. Wang M. H. Wang H. Zhou J.. (2015). Ribosomal proteins and human diseases: pathogenesis, molecular mechanisms, and therapeutic implications. Med. Res. Rev. 35, 225–285. 10.1002/med.21327 25164622 PMC4710177

[B55] Winkler G. S. Balacco D. L. (2013). Heterogeneity and complexity within the nuclease module of the Ccr4-Not complex. Front. Genet. 4:296. 10.3389/fgene.2013.00296 24391663 PMC3870282

